# Telemedicine and E-Learning in a Primary Care Setting in Sudan: The Experience of the Gezira Family Medicine Project

**DOI:** 10.1155/2015/716426

**Published:** 2015-12-29

**Authors:** K. G. Mohamed, S. Hunskaar, S. H. Abdelrahman, E. M. Malik

**Affiliations:** ^1^Department of Family and Community Medicine, University of Taibah, Medina, Saudi Arabia >; ^2^Department of Global Public Health and Primary Care, University of Bergen, Bergen, Norway; ^3^Department of Family and Community Medicine, University of Gezira, Madani, Sudan; ^4^Ministry of Health, Madani, Gezira, Sudan

## Abstract

Information and communication technology (ICT) is progressively used in the health sector (e-health), to provide health care in a distance (telemedicine), facilitate medical education (e-learning), and manage patients' information (electronic medical records, EMRs). Gezira Family Medicine Project (GFMP) in Sudan provides a 2-year master's degree in family medicine, with ICT fully integrated in the project. This cross-sectional study describes ICT implementation and utilization at the GFMP for the years 2011-2012. Administrative data was used to describe ICT implementation, while questionnaire-based data was used to assess candidates' perceptions and satisfaction. In the period from April 2011 to December 2012, 3808 telemedicine online consultations were recorded and over 165000 new patients' EMRs were established by the study subjects (125 candidates enrolled in the program). Almost all respondents confirmed the importance of telemedicine. The majority appreciated also the importance of using EMRs. Online lectures were highly rated by candidates in spite of the few challenges encountered by combining service provision with learning activity. Physicians highlighted some patients' concerns about the use of telemedicine and EMRs during clinical consultations. Results from this study confirmed the suitability of ICT use in postgraduate training in family medicine and in service provision.

## 1. Introduction

The progressive development in the field of information and communication technology (ICT) during the last decades has led to great advances also in the health sector (e-health). There is a global, regional, and national awareness about the importance of e-health. The World Health Organization (WHO) Millennium Development Goals (MDGs) Gaps Task Force has encouraged the governments to increase the use of ICT in the provision of health services in order to increase its efficiency and support the achievement of the MDGs [1]. The WHO Eastern Mediterranean Regional office took a strategic direction to emphasize the importance of using ICT in health, “Leveraging e-Health: Use of ICT in Health in the Eastern Mediterranean Region” [2]. At the national level, many countries (including Sudan) include e-health in their national health plans. However there is a large variation between countries in their commitment and implementation of e-health strategies [3].

The Gezira Family Medicine Project (GFMP) is a collaboration project between the Faculty of Medicine, University of Gezira, and the Ministry of health in Gezira state of Sudan. It aims to improve the quality of primary care health services, through training of family doctors and improving service delivery at this level. GFMP provides a two-year master's program in family medicine since 2010 for the practicing doctors in both urban and rural areas [4]. ICT was implemented in three main areas: telemedicine, e-learning, and the electronic medical records (EMRs).

Telemedicine is defined as the use of medical information exchanged from one site to another via electronic communications to improve a patient's clinical health status [5]. In this paper the term is used to describe online interaction between specialist doctors and the candidates enrolled in GFMP through a videoconference-like meeting. Telemedicine at the GFMP aims to break down the geographical barrier and to increase access to a high quality care at the specialist level to patients all over Gezira state. It was also meant to facilitate the goal of scaling up family medicine training [6]. Although there are some articles evaluating telemedicine in developing countries [7], a literature review in the Eastern Mediterranean region showed that e-health has not yet been well studied [2]. Numerous telemedicine articles are found from developed countries, but in general there has been relatively scarce telemedicine research from developing countries [8].

In this study “e-learning” means the provision of online education using virtual class rooms. It was selected on basis of the great educational opportunities it provides for the students and its enhancement of faculty effectiveness and efficiency [9, 10].

Use of electronic medical records (EMRs) is expected to increase patients' safety and is of high value in effective service provision [11]. Many EMR programs are developed worldwide for both hospital and primary care levels and are sometimes used for specific diseases like HIV in Africa [12] or for follow-up of chronic diseases [13].

This study evaluates the comprehensive experience of using ICT both in education and in service provision at GFMP. It describes the physicians' experience of e-learning as a training method and its role in facilitating the in-service model of training at the GFMP. Administrative data was used to show the actual utilization of ICT in education and practice while questionnaire-based data was used to assess candidates' perception and satisfaction regarding the use of ICT.

## 2. Methods

### 2.1. Study Area

Gezira state lies in the central part of Sudan and has a total population of 3.7 million; about 80% are living in rural areas. Health services are provided through over three hundred health centers which are served by medical doctors or medical assistants (nurse); the second line is rural hospitals or city hospitals. Infectious diseases like malaria represent the major cause of morbidity and mortality in Gezira; however Noncommunicable Diseases (NCDs) are emerging. Internet services are available throughout the state by many companies.

### 2.2. Study Design and Study Population

A cross-sectional observational design was followed to collect administrative and questionnaire-based data. The study targeted the 207 candidates (medical doctors) of the first batch enrolled in the GFMP master program. The number of candidates declined gradually from 207 at the program start to reach 125 candidates at their graduation exam; the main reason was migration to other rich countries. Administrative data collection for the use of telemedicine has targeted all the 207 candidates (from the program start), while questionnaire-based evaluation has targeted the 125 candidates at their graduation.

### 2.3. Data Collection

Registration forms were filled at every telemedicine session. The form included date of the consultation, age and sex of the patient, clinical symptoms, tentative diagnosis, management, and any referral to secondary care, besides the name of the consulting family doctor and consulted specialist doctor. Data from GFMP annual reports for the period from April 2011 (telemedicine start) to December 2012 were used in this paper.

Data collected for EMRs and “e-learning” was obtained from administrative data and exam results' data. The candidates' use of the ICT tools was a part of their evaluation at the family medicine exams, which included the number of initiated and opened electronic patient files (quantity), the comprehensiveness of the data in the files (quality), the candidates participation in telemedicine activity (number of consultations), and the candidate's participation in the online e-learning activities.

Candidates' own evaluation of the use and effect of the different ICT elements, that is, telemedicine, EMRs, and e-learning, was registered through an anonymous questionnaire filled by them at the end of their study period of the master's program. We used a six grades' scale: “Strongly agree” (6), “Somewhat agree” (5), “Agree” (4), “Disagree” (3), “Somewhat disagree” (2), and “Strongly disagree” (1). A total of 113 candidates responded to the ICT self-evaluation questionnaire out of the total number of 125 graduating candidates, a response rate of 90.4%.

### 2.4. Telemedicine Program Implementation

At the start, all of the 207 trainees were provided with computer laptops with an in-built camera for telemedicine and training purposes; they were also provided with free internet service; all was paid by Gezira state government. Two telemedicine studios were established and equipped with computers, LCD screens, cameras, microphones, Internet, and telephone lines.

The software program ooVoo [14] was used to connect family doctors (together with the patient) at the health centers all over Gezira state with the specialists at the GFMP headquarter in Madani town (capital of the state). They communicated by means of voice, picture, and chatting in a videoconference-like setting, allowing the family doctor to show the specialist some clinical signs on the patient such as skin manifestations or some investigations like ECG. A weekly scheduled program was set for specialist doctors in telemedicine activities, provided to the candidates so that they could communicate with the needed specialist at the scheduled times.

### 2.5. Electronic Medical Records' Implementation

Before the start of the GFMP, there were few health centers in Gezira state that were not using medical records at all, even as paper notes. GFMP developed a specially designed electronic medical record program called Family Clinic. The program copes with local needs and includes the ICD10 classification of diseases.

### 2.6. E-Learning Implementation

The same studios used for telemedicine were also used for educational activities. Cisco-WebEx software [15] was installed in the computers of the candidates and at the studios; it is used to connect all candidates with the lecturer. Candidates could see, hear, and interact with the lecturer who could share his desktop screen with the candidates to show them pictures, slide presentations, videos, or text documents. Full interaction through online discussions between the candidates and the lecturer was possible. Any trainee could also take the “host” role and present a tutorial or other activity to the other trainees and the lecturer. It was also possible to download the online activities later from the website of the GFMP; some candidates preferred to attend the lectures on portable devices, especially when there were problems with internet connectivity.

### 2.7. Statistical Analyses

The IBM SPSS program version 21 (supplier: IBM corp., Armonk, NY, USA) was used for data management and statistical analyses. Results are presented as descriptive statistics with means, proportions, and percentages.

### 2.8. Ethical and Privacy Approvals

The study was reviewed and approved by the Ethical Review Committee at the Ministry of Health, Gezira state, Sudan. The study proposal was also approved by the Regional Committee for Medical and Health Research Ethics, Western Norway. Privacy issues and patients' file management related to the scientific evaluation were also approved by the Norwegian Data Protection Official for Research.

## 3. Results

### 3.1. Telemedicine

**Figure 1:**
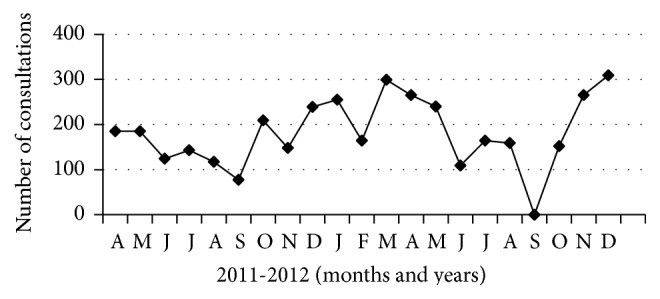
Monthly distribution of consultations (*N* = 3808) from April 1, 2011, to December 31, 2012.

The telemedicine activity at the GFMP started on April 1, 2011, almost six months after the start of the program. A total of 3808 telemedicine consultations have been registered in the period from April 1, 2011, to December 31, 2012. The monthly distribution of consultations is shown in Figure 1. The mean number of consultations per month was 181, with a slight monthly increase during the first year, reaching its maximum of 299 consultations in March 2012; this was followed by a large decrease to a minimum of zero consultations in September 2012 (due to internet disconnection), followed again by an increase reaching its maximum of 309 consultations in December 2012.

**Figure 2:**
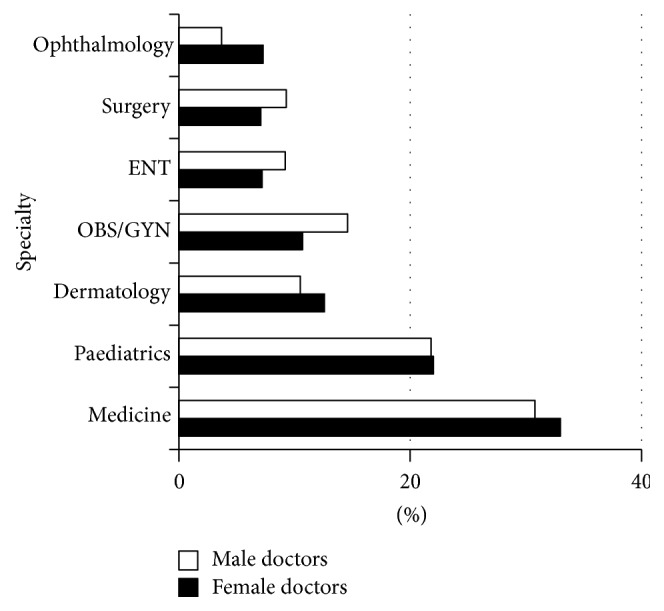
Distribution (%) of telemedicine consultations by specialty and doctor's gender (*N* = 3749). ENT: ear, nose, and throat; Obs/Gyn: obstetrics and gynaecology.

The majority of the consultations (*N* = 2763, 74%) were held by female doctors (mean 31 consultations per female doctor), compared with 8 per male doctor. Almost one-third of the consultations were related to the discipline of internal medicine (32.6%) followed by paediatrics (21.8%). Male doctors used to have more consultations in obstetrics and gynaecology, surgery, and ENT, while female doctors used to consult specialists in ophthalmology, dermatology, and internal medicine in a higher proportion than men (Figure 2).

The vast majority of telemedicine consultations were done by doctors working in Madani locality (2234 consultations, mean 35 per doctor), followed by El-Kamlin locality (469 consultations, mean 19 per doctor). Least consultations were registered from East-Gezira locality (55 consultations, mean 3 per doctor).

#### 3.1.1. Trainees' Evaluation of the Use of Telemedicine

Findings from the candidates' evaluation of the use of telemedicine are shown in Table 1. All respondents agreed to varying degrees on the high importance of telemedicine to their patients' care. They also agreed that it was a good training method for them. The vast majority (89%) agreed that patients were satisfied with telemedicine. The majority (81%) usually preferred taking telemedicine consultations with the patient being in the office. However, almost one-fifth of the candidates agreed (in varying grades) that patients might lose the confidence in their family doctors if telemedicine consultations were used. In general, males demonstrated more positive attitude, compared with females, regarding several aspects of telemedicine, although this was not statistically significant (Table 1).

### 3.2. Electronic Medical Records (EMRs)

A total of 165993 new patients' EMRs were created by the study subjects, 125 candidates, mean of 1328 files per doctor (maximum 5470, minimum 104, and median 1178). The mean number of files opened by male doctors was 1413 (*N*: 49 male doctors), while the mean for female doctors was 1273 (*N*: 76 female doctors).

#### 3.2.1. Trainees' Evaluation of the Use of Electronic Medical Records, EMRs

Almost two-thirds of the respondents strongly agreed that EMR is important for the care of their patients, while a third agreed or somewhat agreed. On the other hand a fifth strongly agreed, and more than a half agreed or somewhat agreed that patients are not happy when the family doctor was using a computer during consultation. The majority of respondents stated that they register all or most of their patients in EMRs. Males reported a more positive attitude (statistically not significant) than females regarding the use of the EMR (Table 1).

### 3.3. E-Learning

Administrative data showed that 240 online lectures and 29 meetings were organized using the software program Cisco-WebEx in the period from May 2011 to October 2012. Family medicine constituted almost 38% of the presented lectures (*n*: 90), internal medicine 23% (*n*: 55), paediatrics 15% (*n*: 37), obstetrics and gynaecology 8% (*n*: 19), and surgery 8% (*n*: 18), and the rest was distributed between other medical disciplines.

#### 3.3.1. Trainees' Evaluation of the Use of E-Learning

Almost two-fifths of the respondents “strongly agreed” that online lecture was a good teaching method. One-half of the candidates either “agreed” or “somewhat agreed,” while 9% “disagreed,” “somewhat disagreed,” or “strongly disagreed” (Table 1). There was a large variation in the evaluation of the candidates regarding the easiness of combining work with the training activities; 28.6% reported degrees of disagreement, while 71.4% reported degrees of agreement. The difference between males and females is more prominent in the e-learning component although it did not reach statistical significance level (*P* = 0.072 and *P* = 0.070); thus males had a tendency of more positive attitude to the utilization of e-learning.

## 4. Discussion

A successful integrated utilization of ICT at the GFMP in Sudan is shown in this study. A high number of telemedicine consultations were achieved, with high satisfaction among the candidates. EMRs were introduced in this area for the first time, and more than 165,000 patient files were opened in a period of two years, reflecting a great need for such innovative solutions in primary care settings in developing countries. Using ICT at the GFMP has also contributed to universal health coverage and partially reduced the problem of brain drain which is a recognized challenge in developing countries [16]. GFMP recruited 207 doctors to work at primary health care facilities, where 84 health centers had never been served by a physician before [4]. This finding is in agreement with that of a study from Mali (2012) which reflected the positive significant influence of ICT on the recruitment and retention of health care professionals [17].

This successful use of telemedicine activity follows a global trend of positive experience in using telemedicine [18], which is also documented in developing countries [19–21]. A survey from the WHO in 2014 revealed encouraging findings in many countries regarding e-health and innovation in women's and children's health [22].

The monthly variation in the number of telemedicine consultations represents the expected challenges facing ICT implementation, including technical problems, academic obligations (exam periods), and sometimes economic constrains where the project was unable to afford internet fees. Discipline based variation in telemedicine utilization was prominent; this can be due to the spectrum of common diseases in the area. Telemedicine activity was not used in emergency cases at the GFMP, in contrast to other experiences where telemedicine has been used only in emergency and disaster settings [23]. The candidates were highly satisfied with telemedicine and found it as an effective pathway to communicate quickly and effectively with the physicians working in second-line care.

The huge number of electronic records establishes a data pool that is suitable for research as well as for patients' care. The variation in the number of registered patients between physicians could mainly be attributed to the size of population in the catchment area, in addition to the activity of each health center. The physicians' high activity in using the EMR system was influenced by the Workplace Based Assessment (WBA) method of evaluation in their Family Medicine Master's exam; both the quantity (number of files) opened by the candidate and the quality (comprehensiveness) of the files were evaluated in the exam. A systematic review of the impact of WBA on doctors' education and performance showed positive performance changes [24]. Physician's attitude towards the use of computer during consultation provided similar results to a study done in the United Arab Emirate (UAE), where concerns including effectiveness of patients' communication were also highlighted [25]. Other studies assessing patients' satisfaction with the use of computers during consultation revealed general satisfaction [26–28].

E-learning was used at the GFMP in a blended way, including both face to face teaching and e-learning. A review article compared the use of blended e-learning methods with the traditional approaches in medical education in resource constrained countries. It revealed either better-promising results or no statistically significant differences between the two approaches in the majority of studies [9]. A study from Iran comparing the effect of lecture and blended teaching methods on students' learning and satisfaction showed higher satisfaction among students using blended method [29].

*Study Limitations*. Several limitations should be recognized. Firstly, reduction of the number of enrolled candidates (207 to 125) within the 2-year training period, due to brain drain to rich countries. Secondly, information on patients' perception on the use of the EMR and telemedicine utilization during consultation was obtained from the study subjects (family physicians), instead of the patients themselves. This study collected physicians' perceptions about patients' feelings generally, not at an individual patient's basis. Finally, social desirability bias of self-administered questionnaires was reduced by blinding the name of the participants. In addition, administrative data reflects the real practice and could approve or disapprove the physicians' perception. In this case the high use of ICT tools was in harmony with the high satisfaction reported by the participants.

## 5. Conclusion and Recommendations

GFMP's experience in utilizing ICT in a comprehensive way, including telemedicine, EMRs, and e-learning, is rather unique in a developing low-resource country setting. Results from this study show how ICT implementation in primary care can participate in achieving important health care goals like universal coverage, accessibility, decreasing brain drainage, and increasing the training's capacity in the health sector. Although the project was politically and financially supported on a national level, the successful implementation was not without barriers and challenges, such as internet coverage and technical problems. The issue of sustainability deserves to be considered and further research is needed to explore the impact of using ICT on health indicators in the project.

## Figures and Tables

**Table 1:** Doctors' evaluation of the ICT tools used at the GFMP (using Chi square test) (*n* = 113).

Statement text	Distribution (%) of doctors' evaluation						Mean values			*P*
	Strongly agree (6)	Somewhat agree (5)	Agree (4)	Disagree (3)	Somewhat disagree (2)	Strongly disagree (1)	Total	Male	Female	
*Telemedicine*										
(1) Telemedicine is of high benefit for my patients	62.8	11.5	25.7	0	0	0	5.37	5.43	5.31	0.839
(2) It is a good teaching tool for family doctors	63.4	6.2	29.5	0.9	0	0	5.32	5.41	5.26	0.714
(3) I use it usually when the patient is in my office	25.2	15.3	40.5	14.4	0.9	3.6	4.39	4.41	4.37	0.924
(4) My patients are usually satisfied with telemedicine	28.2	20.9	40.0	6.4	0.9	3.6	4.58	4.74	4.48	0.571
(5) The patients miss the confidence on family doctor when telemedicine is used	6.4	6.4	7.3	48.6	5.5	25.7	2.83	2.95	2.74	0.516
*Electronical medical records (EMR)*										
(1) The EMR system is highly important for my practice	65.2	4.5	27.7	0.9	0	1.8	5.29	5.40	5.22	0.365
(2) The patients are not happy when I write on the computer during consultation	18.6	16.8	38.1	14.2	3.5	8.8	4.06	4.05	4.07	0.559
(3) I register all my patients on the EMR	27.0	18.9	26.1	18.9	8.1	0.9	4.35	4.47	4.28	0.695
*E-learning*										
(1) Online lectures are a good teaching method	40.5	15.3	35.1	5.4	2.7	0.9	4.83	5.23	4.57	0.072
(2) It was easy to combine the work with the training activities	23.2	11.6	36.6	16.1	4.5	8.0	4.09	4.45	3.85	0.070
